# Soil–Plant Relationships in Soybean Cultivated under Crop Rotation after 17 Years of No-Tillage and Occasional Chiseling

**DOI:** 10.3390/plants11192657

**Published:** 2022-10-10

**Authors:** Gustavo Ferreira da Silva, Ana Paula Oliveira Matusevicius, Juliano Carlos Calonego, Larissa Chamma, Bruno Cesar Ottoboni Luperini, Michely da Silva Alves, Hugo Mota Ferreira Leite, Elizabete de Jesus Pinto, Marcelo de Almeida Silva, Fernando Ferrari Putti

**Affiliations:** 1 Department of Crop Science, School of Agriculture, São Paulo State University (UNESP), Botucatu 18610-034, Brazil; 2 Multidisciplinary Center, Federal University of Acre (UFAC), Cruzeiro do Sul 69980-000, Brazil; 3 Health Sciences Center, University of Recôncavo of Bahia (UFRB), Santo Antônio de Jesus 44574-490, Brazil; 4 Department of Biosystems Engineering, School of Sciences and Engineering, São Paulo State University (UNESP), Tupã 17602-496, Brazil

**Keywords:** cover crops, *Glycine max*, plant physiology, root development, soil physics, soil water

## Abstract

No-tillage cover crops contribute to better soil quality, being able to replace mechanized tillage management. This observation can only be made after several years of adopting conservationist practices and through research on soil–plant relationships. The objective of the research was to verify the relationship between the production components, physiological, root development, and physical-hydric properties of the soil in the yield of soybean grown in succession to different cover crops or with soil chiseling. The experiment was carried out in a randomized block design with four replications, comparing the cultivation of sunn hemp (*Crotalaria juncea*) and millet (*Penninsetum glaucum* L.) as cover crops and a treatment with soil chiseling. The evaluations were carried out during soybean (*Glycine max* L.) cultivation in the 2019/20 summer crop, that is, after 17 years of experimenting started in 2003. Rotation with sunn hemp increased soybean yield by 6% and 10%, compared with millet rotation and soil chiseling. The species used in crop rotation in a long-term no-tillage system interfere with the physical and water characteristics of the soil, affecting the physiological responses and soybean yield. The rotation with sunn hemp offers greater water stability to the plants and provides greater soybean yield in succession. Future research that better addresses year-to-year variation, architecture, and continuity of pores provided by crop rotation, and evaluations of gas exchange, fluorescence, and activities of stress enzymes in soybean plants may contribute to a better understanding of soil–plant relationships in long-term no-till.

## 1. Introduction

No-tillage system (NT) represents a milestone in water and soil conservation in agricultural systems due to its precepts, such as not tilling the soil, rotating crops, and keeping the soil covered with straw [1,2].

Among these precepts, crop rotation plays a fundamental role in the success of NT through straw production and diversification of root systems in the soil [1]. In addition to reducing erosion and increasing soil moisture retention capacity, the permanent soil cover promoted by NT reduces surface runoff and surface water evaporation [2]. In addition to these factors, the higher organic matter content in the surface layers contributes to higher porosity, favoring a higher infiltration rate [3,4].

Another important point is the large roots production by cover crops, which end up exploring a large volume of soil profiles and are capable of altering the physical-hydric soil properties and, consequently, the productivity of the crop grown in succession [5].

The cover crops roots are important tools for manipulating the soil structure to obtain beneficial conditions for successor crops growing [6,7,8]. Research shows that plants with thick, deep roots and those with thin, voluminous roots are effective at improving soil structure, creating paths while growing, and producing biopores after root death [8,9,10,11,12]. These biopores act on liquid–gas transport, which improves soil aeration and reduces surface runoff and erosion [13,14,15]. In addition, biopores can provide preferential pathways for root growth of successor plants, as they provide spaces with low mechanical resistance and high concentrations of oxygen, favoring root respiration and growth in depth, thus contributing to greater soil exploration in search of nutrients and water [11,16,17].

Despite the potential of cover crops to improve soil structure, mechanical management with soil chiseling is widely used to alleviate soil compaction [18], however, many studies question this practice because it produces ephemeral results [8,19,20,21,22].

There is already research addressing the effects of cover crops and soil chiseling about soil physical, chemical, and biological properties and about crop productivity [5,8]; however, this subject requires new research that present the results obtained in long-term experiments. In addition, some studies show isolated effects on soil or productive components of crops without considering the soil–plant relationship [2,5,8].

In this way, knowing that the use of cover crops and soil chiseling affect soil structuring, and considering that soybean is one of the main agricultural crops susceptible to environmental conditions [23] and soil management [24], this research hypothesizes that the use of different cover crops in the crop rotation in long-term NT and soil chiseling can affect the physical-hydric properties of the soil, as well as the physiology of the plant, resulting in alteration in soybean development and yield.

Thus, the objective of this research was to verify how physical and hydric properties of the soil, as well as the yield, physiological, and root development variables interrelate and consequently affect the yield of soybean grown in a long-term no-tillage system with different crop rotation and with soil chiseling.

## 2. Results

From the correlation matrix between the pairs of each variable group (yield components, physiological traits, root development, soil physics, and soil water), the eigenvalues and their respective eigenvectors were obtained for the analysis of principal components.

The first principal component has already explained more than 70% of the variance for all variable groups (Table 1). Therefore, only the first component was considered for exploiting results.

**Figure 1:**
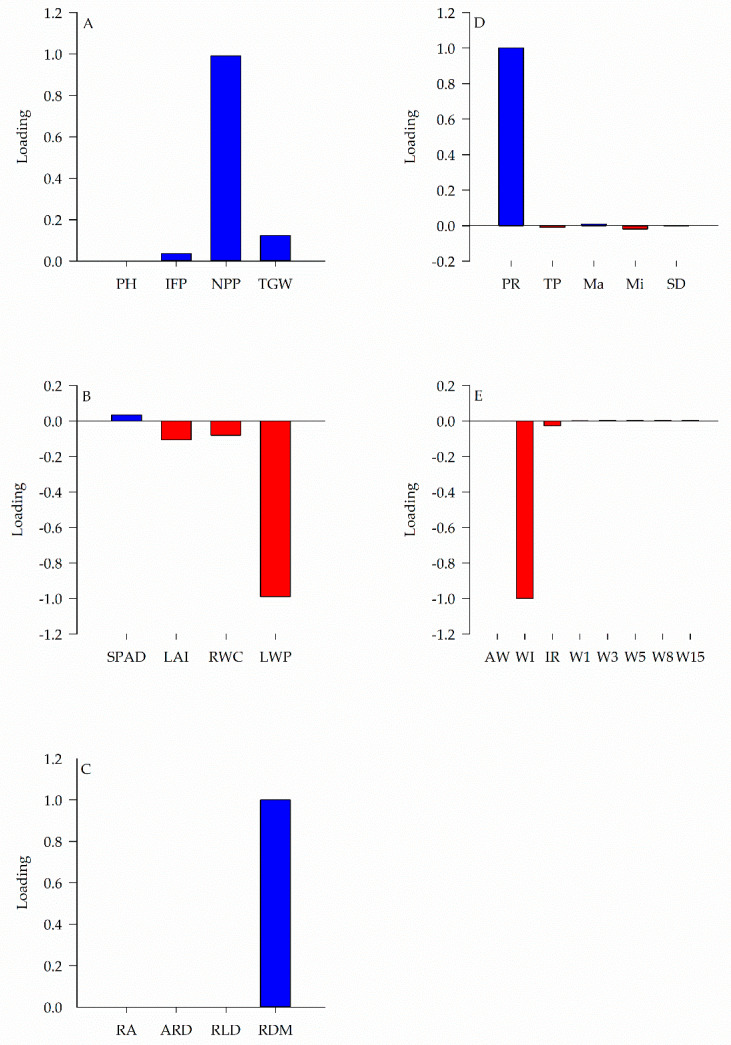
Loadings of variable in yield components (**A**), physiological traits (**B**), root development (**C**), soil physic (**D**), and soil water (**E**). PH: plant height; IFP: insertion of the first pod; NPP: number of pods per plant; TGW: thousand grain weight; SPAD: index SPAD; LAI: leaf area index; RWC: leaf relative water content; LWP: leaf water potential; RA: root area; ARD: average root diameter; RLD: root length density; RDM: root dry matter; PR: soil penetration resistance; TP: soil total porosity; Ma: soil macroporosity; Mi: soil microporosity; SD: soil density; AW: available water capacity; WI: accumulated water infiltration; IR: basic infiltration rate; W1: water stored one day after rain; W3: water stored three days after rain; W5: water stored five days after rain; W8: water stored eight days after rain; W15: water stored 15 days after rain. Blue color indicates positive loading variable; and the red color indicates a negative loading variable.

Among all the variables analyzed, the number of pods per plant (NPP), thousand grains weight (TGW), leaf water potential (LWP), leaf area index (LAI), the relative water content in the leaf (RWC), root dry matter (RDM), soil penetration resistance (PR), accumulated infiltration(WI), and the basic rate of water infiltration into the soil (IR) (Figure 1), were the characteristics that most explain the respective components, with loadings of 0.99, 0.12, −0.99, −0.11, −0.08, 1.00, 1.00, −1.00, and −0.027, respectively.

**Figure 2:**
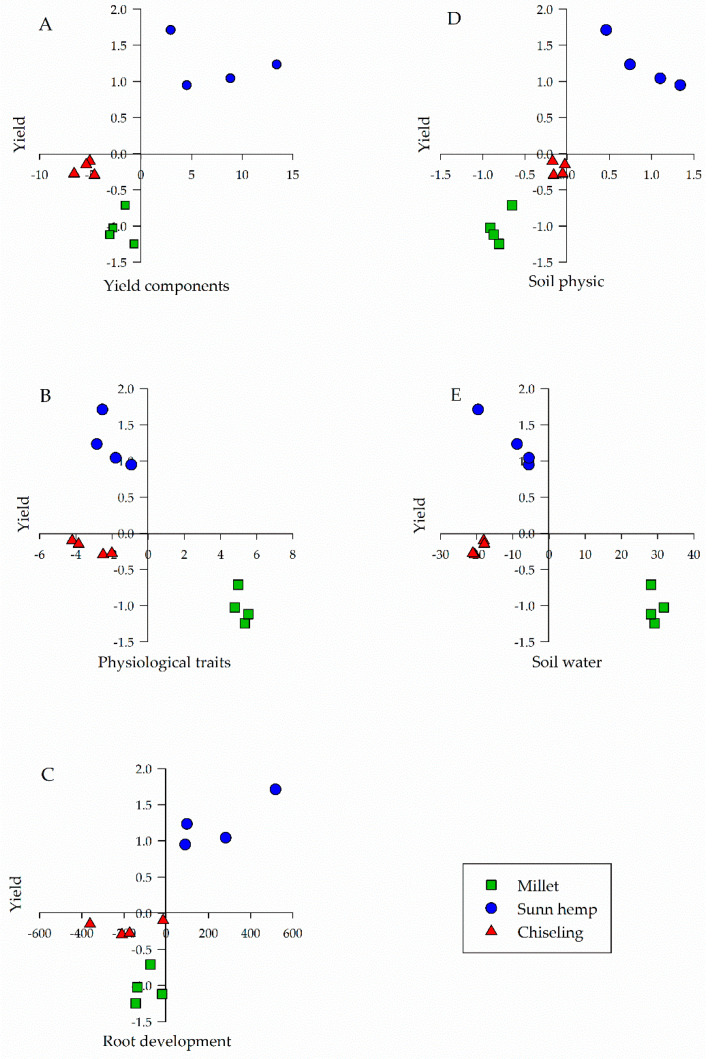
Dispersion of observations in principal component 1 of yield components (**A**), physiological traits (**B**), root development (**C**), soil physics (**D**), and soil water (**E**) variables.

Through two-dimensional dispersion of treatments, there was a difference between the species used in crop rotation and soil chiseling for all groups of variables. Plants grown under a no-tillage system (NT) with sunn hemp in crop rotation during the spring season showed a higher yield than when NT with millet rotation or with soil chiseling (Figure 2).

The highest soybean yield, observed in crop rotation with sunn hemp during the spring season (3.900 kg ha^−1^), was associated with plants with a greater number of pods and with greater thousand grains weight (Figure 2A); greater leaf area index, water content and water potential in the leaf (Figure 2B); greater root dry matter (Figure 2C) and soil penetration resistance (Figure 2D); and greater infiltration and the basic rate of water infiltration into the soil (Figure 2E).

**Figure 3:**
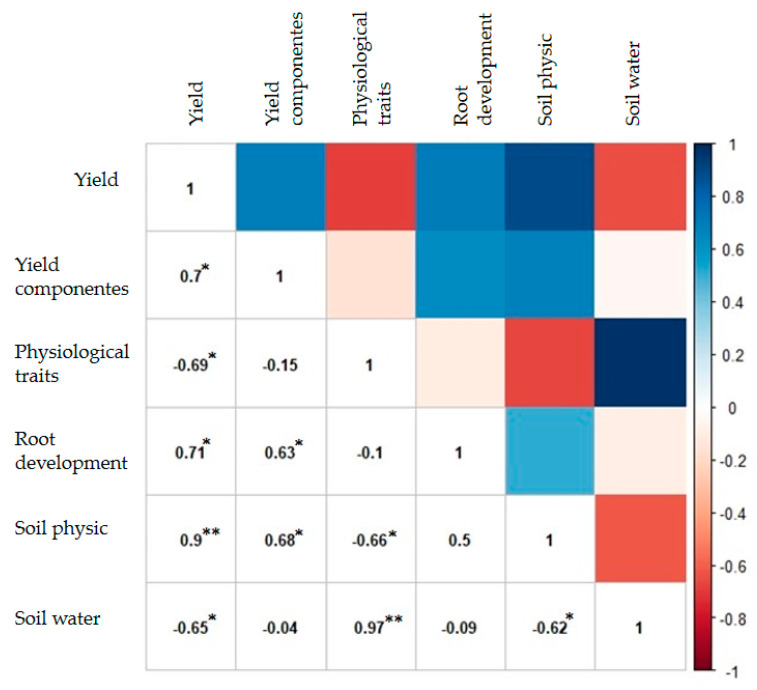
Pearson correlation between the first principal component of each variable group. ** and * indicates significant correlation at *p* < 0.01 and *p* < 0.05, respectively.

Analyzing Pearson’s correlation analysis (Figure 3), it was observed that variables of yield components (0.7), physiological traits (−0.69), root development (0.71), soil physics (0.9), and soil water (−0.65) showed a significant correlation with soybean yield.

Regardless of the species used in crop rotation in NT or soil chiseling, yield showed a significant correlation with all sets of variables (yield components, physiology traits, root development, soil physic, and soil water) (Figure 3). It is noteworthy that the higher the number of pods per plant and the thousand grains weight, the better the soybeans effective response (Figure 1 and Figure 3). The higher leaf area index, relative water content, and water potential in the plant are also associated with greater root dry matter, greater soil penetration resistance, and more significant infiltration and rate infiltration of water in the soil, which also contribute to higher yield (Figure 1 and Figure 3). However, the highest yield was observed in a rotating system with sunn hemp (Figure 2).

There was also a significant correlation between the yield components and those of soil physic and soil water. The physiological trait component was also correlated with soil physics and soil water. It also showed a significant correlation of soil water with soil physics (Figure 3).

## 3. Discussion

The greater production of pods per plant and thousand grains weight obtained in the system involving sunn hemp in the crop rotation for 17 years led to an increase in yield of approximately 6% and 10%, in comparison with the rotation using millet or soil chiseling (Figure 1A and Figure 2A). The best response of yield components in the treatment with sunn hemp is related to changes in physical properties and root development of soybean in this treatment. Such association can be confirmed by the direct and significant correlation between these groups of variables (Figure 3).

Higher values of PR in NT generally do not harm plant development due to greater continuity of pores, making porosity more efficient in liquid and gaseous transport and favoring root growth [25,26]. Considering that the roots of leguminous plants are more aggressive in breaking up compacted layers compared to grasses [8], sunn hemp probably promoted greater formation of biopores in the soil than millet, in a field managed with these same crops for 17 consecutive years.

Management systems without soil disturbance generally present greater nutrient accumulation in soil, mainly in the superficial layer, where there is a greater amount of soybean roots [27,28], favoring the nutrients absorption, contributing to greater production of pod and grain weight [29], as in this research results for the treatment with sunn hemp.

In the treatment with millet, there is also no soil disturbance. Despite having similar nutrient availability to the treatment with sunn hemp, it has greater soil acidity, which impairs the absorption of nutrients [30]. Crop rotation with legumes favors an increase in soil pH compared to grasses since N_2_ fixation produces less H^+^ than NH_4_^+^ assimilation by the plants, contributing to the reduction of rhizosphere acidification [31,32].

Based on plant physiological responses, it was noted that soybean had higher water availability in the NT with sunn hemp rotation, explained by higher leaf area index, relative water content, and leaf water potential (Figure 1B and Figure 2B). This behavior can also be observed by the direct and significant correlation between soil water components and physiological characteristics (Figure 3), indicating that the greater the water storage in the soil, the greater the water content in the plant. This physiological behavior resulted in higher soybean yield in NT (Figure 2B).

Regarding the soybean physiological responses, this component showed a negative correlation with yield (Figure 3). However, it is important to notice that the main variables of this group showed a negative loading (Figure 1B). Thus, in the treatment with sunn hemp, higher yield was associated with higher leaf area index, relative water content, and leaf water potential (Figure 2B). In the millet rotation, these parameters were lower, resulting in lower yield (Figure 2B). Moreover, in the treatment with soil chiseling, even with physiological behavior similar to the management with sunn hemp, it did not result in higher yield (Figure 2B).

The treatment with millet showed low water content in the soybean plant (Figure 2B), which may be associated with lower soil exploitation by the root system [33], since it had low RDM production (Figure 2C). Meanwhile, the treatment with soil chiseling, even with high water content in the plant, did not result in high yield (Figure 2B). It should be noted that the evaluations of this research were carried out in the 2017/18 harvest, 16 months after soil chiseling; therefore, it is likely that the beneficial effects of chiseling on the soil physical properties were no longer present, considering that its effects are temporary on soil structure and plant development, generally not exceeding one year [8,19,20]. Thus, the higher water content in the plant was not enough to result in higher yield.

The difference between treatments with sunn hemp and millet in physiological responses may be associated with the quantity and quality of straw formed by each crop and with the different root systems these species present [34]. The rotation with cover crops improves the soil physical quality [35]. In contrast, the rotation with sunn hemp increases the soil macroporosity when compared to the rotation with millet [8]. This also explains the lower infiltration and rate of water infiltration into the soil in the millet treatment (Figure 2E), which can also be verified by the negative correlation between soil water and yield (Figure 3). In this sense, it is worth noting that the variables with the highest loadings in soil water presented negative loads (water infiltration and infiltration rate) (Figure 2E). Thus, the smallest amount and rate of water infiltration into the soil, with millet rotation, reduced soybean yield.

The greater macroporosity favors root growth, water infiltration, and oxygen diffusion in the soil profile. In low oxygen availability, the roots produce ethylene, which is toxic to plants [36]. In soybeans, lack of oxygen impairs symbiotic nitrogen fixation, decreasing yields [37].

In general, the higher soybean yield in succession to the sunn hemp can be explained by the association with physiological characteristics, root development, soil physics, and soil water. However, these results were based only on evaluations carried out 17 years after the experiment implementation. It is possible that there are some variations between these parameters between seasons, mainly due to climatic conditions, which directly interfere in the plants development. Therefore, new studies that evaluate these characteristics year to year can contribute to a better understanding of the water, soil, and plant relationships that are established in long-term management systems.

The higher PR, observed in the sunn hemp rotation system, did not affect the water infiltration in the soil thus contributing to greater water availability to plants. With a greater water supply, the plants did not need to develop roots in depth due to the water supply provided by the system, reducing the energy expenditure with root development and also contributing to the best water content in the plants [24,38,39]. Thus, rotation with sunn hemp in long-term NT was characterized as a production system less vulnerable to dry spells and drought.

Plants grown under soil water deficit conditions develop adaptive mechanisms to survive under these conditions [40]. Stomatal closure is the first line of defense against dehydration [41]. The lack of water and consequent stomatal closure results in the exposure of excess energy in the plant, which, if not safely dissipated, can cause excitation in the reaction center of the PSII photosystem, resulting in photoinhibition [42], initiating the production of H_2_O_2_ which, consequently, leads to the activation of antioxidant metabolism [43].

Thus, the appropriate choice of species to use in a crop rotation system in NT can lead to greater water availability [8,24]. Understanding the dynamics of water in the soil becomes essential for correctly planning agricultural activities, with water being the factor that most affects crop yield [44].

There are several factors related to the water storage capacity in the soil and its availability to plants. However, one of the main factors is soil management, as it modifies the soil physical properties associated with the structure, such as water availability, aeration, and resistance to root growth, which are directly related to plant development [24].

Compared to soil chiseling treatment, the positive effect of sunn hemp (grown for 17 consecutive years in the spring season) on soil water dynamics is also associated with the presence of straw on the soil, as it reduces the impact of raindrops on the surface, decreases surface runoff and evaporation surface water [3,45]. In addition to these factors, the higher organic matter content in the surface layers contributes to the greater structuring and soil porosity, favoring a higher rate of water infiltration [46].

## 4. Materials and Methods

### 4.1. Site Description

The field experiment was conducted in Botucatu, SP, Brazil, situated at 22°49′ S and 48°25′ W, at an altitude of 780 m, on a Typic Rhodudalf [47], classified as a clayey texture. The soil chemical [48] and textural [49] properties are presented in Table 2.

**Figure 4:**
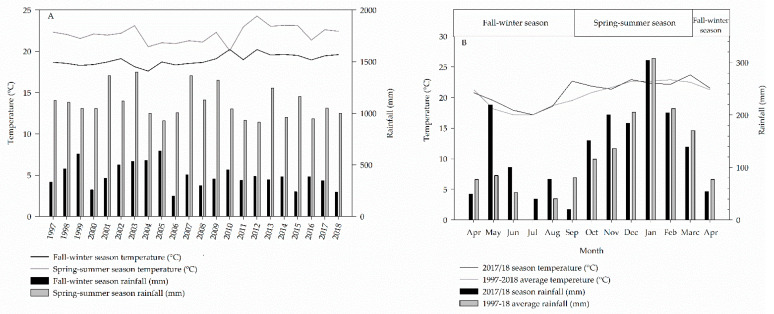
Means temperature and rainfall in fall–winter and spring–summer seasons since experiment implementation (1985–2018) (**A**), and means monthly temperature and rainfall between the years 1985 and 2018 and during the 2017/18 season (**B**).

The climate is CWa type, according to the Köppen classification, which means mesothermal climate with dry winter, with a mean annual rainfall of 1450 mm [50]. Means temperature and rainfall between the years 1997 and 2018 and during 2017/18 season are shown in Figure 4, respectively.

### 4.2. History and Experimental Design

The experiment began in 2003 with triticale (*X Triticosecale* Wittmack) cultivated in fall–winter in the total area; millet (*Penninsetum glaucum* L.) and sunn hemp (*Crotalaria juncea* L.) as cover crops in the spring, plus a treatment with fallow in this season; and soybean was cultivated in the total area in the summer season. The management history of the experiment is shown in Table 3. Where no cover crop was cultivated in spring, soil chiseling was performed every three years from 2003, i.e., it was chiseled in the years 2003, 2009, 2013, and 2016.

The experimental design was a randomized complete blocks with three treatments and four replications. Treatments consisted of the different cover crops in spring (millet and sunn hemp) or soil chiseling was followed by fallow in the spring. The plots were delimited with 40 m^2^ (8 × 5 m), with 4 m spacing between plots and between blocks.

Mechanical chiseling was carried out using a chisel plough with seven shanks set on two parallel bars and spaced 0.60 m from each other within the bars resulting in an effective 0.30 m between drill spacings. The shanks were inclined forward forming a 25° angle and the effective action depth was around 0.30 m. A cylinder was attached to the equipment to break up the biggest clods and smooth the soil surface in order to avoid harrowing.

### 4.3. Management and Analysis of Soybean Plant

In late spring, at preflowering stage of cover crops, the plants were chemically desiccated with glyphosate, and residues were left on the soil surface. The soybean was sown soon thereafter on 8 December 2017, with cultivar TMG 7062 IPRO, using 0.45 m spacing between lines, aiming at the density of 300 thousand plants ha^−1^. Seeds were treated with fungicide Carboxin + Thiran, insecticide Tiametoxam, inoculant *Bradyrhizobium* sp., and micronutrients Co e Mo. The sowing fertilization was conducted with 60 kg ha^−1^ of K_2_O and 60 kg ha^−1^ of P_2_O_5_, using KCl and single superphosphate, respectively. The harvest was carried out 111 days after sowing.

#### 4.3.1. Physiological Traits

The physiological traits were evaluated when the soybean plants were in the full bloom stage (R2). The relative chlorophyll content was obtained through the SPAD index, using the portable chlorophyll meter SPAD-502 (Minolta Corp., Ransey, NJ, USA) in 10 plants per plot, evaluating the third trefoil from the apex.

To determine the leaf area index (LAI), all plant leaves were collected at 0.5 m, and then analyzed in a bench leaf area integrator (LICOR, model LI-3100C, Lincoln, NE, USA). The LAI was calculated with the ratio of the total plant leaf areas (m^2^) per unit of land (0.225 m^2^) available for plants [51,52], according to Equation (1):

LAI = total leaf area/soil surface area(1)

To obtain the leaf relative water content (RWC), five plants were analyzed, and two leaf discs (0.69 cm^2^ each) were collected from the third trifoliolate leaf (apex to the base) of each plant and the fresh tissue mass (Wf) was determined in analytical balance. After that, the samples were rehydrated in distilled water for 24 h, to obtain the turgid mass (Wt), using paper towels to extract the excess water. The dry mass (Wd) was obtained after the discs remained in an oven with forced air circulation at 80 °C for 48 h. The equation of [53] obtained RWC values:

RWC = [(Wf − Wd) × (Wt − Wd)^−1^] × 100 (2)

The leaf water potential (Ψw) was obtained using a Scholander chamber (Soil Moisture Equipment, model 3005, Santa Barbara, CA, USA). The measurements were taken in the hottest period of the day, between 12:00 and 14:00 h, so the lowest values of leaf water potential are observed. Ψw was determined at the end (tip) of the third trefoil (direction from the apex to the base), where pressure was applied until exudation occurred through the cut made in the leaf petiole.

#### 4.3.2. Root Development

Root analysis was performed at the full bloom stage (R2) of soybean. Soil samples were collected with auger hole in the depth of 0.00–0.40 m, with four subsamples per depth to compose a sample.

After collection, the soil portions containing the roots were washed on a 1 mm sieve, placed in a container containing 30% alcohol and 70% water and stored in a refrigerated environment. Subsequently, the samples were subjected to an optical reading scanner at a resolution of 250 dpi, and the images obtained were analyzed with the “Win Mac Rhizo” program to determine the root length density (root cm soil cm ha^−3^), root area (root cm^2^ soil cm^−3^), and average root diameter (mm). Afterward, the samples were placed in paper bags and dried in a forced aeration oven at 60 °C for 48 h to determine the dry mass.

#### 4.3.3. Yield Components

When the crop reached the phenological R9 stage, the plant height, and the size of insertion of the first pod and the number of pods per plant in 50 plants of each experimental unit were evaluated. Thousand grains weight was also assessed, according to [54].

#### 4.3.4. Yield

The soybean yield estimate was performed after the grains’ physiological maturity, harvesting the plants from the useful fields (4.5 m^2^), discarding the borders, and the water content of the grains was corrected to 130 g kg^−1^, according to Equations (3) and (4).

M = [(WGW − DGW)/WGW] × 1000(3)

where M is the grain moisture (g kg^−1^); WGW is the wet grain weight (g); and DGW is the dry grain weight (g).

Wc = [(130 × WGW)/M](4)

where Wc is the grain weight corrected (g kg^−1^); WGW is the wet grain weight (g); and M is the grain moisture (g kg^−1^).

### 4.4. Soil Analysis

#### 4.4.1. Soil Water Storage

The soil water content was assessed using tubes for moisture readings using a capacitance probe (model Diviner^®^, Sentek Pt Ltd., Stepney, SA, Australia). Water content monitoring was carried out from the surface to 0.40 m in depth (0.10 m range), with readings at one, three, five, eight, and 15 days after rain (DAR); for this evaluation, rains above 10 mm were considered. Stored water values (SW) were the result of the sum of the humidity values up to the depth of 0.40 m in each experimental unit, and for each day of reading the average of SW was made.

#### 4.4.2. Soil Physical Properties

At the time of root collections, a soil penetration resistance test (PR) was carried out at three points per plot using the Impact Penetrometer (model IAA/Planalsucar–Stolf, Piracicaba, SP, Brasil).

For the assessment of soil density (Sd), macroporosity (MP), microporosity (mp), total porosity (TP), field capacity (FC), and permanent wilting point (PWP), two samples of soil were analyzed with undeformed structure at each depth, using volumetric rings, by the trench methods [45,55,56]. With the values of FC and PWP, it was possible to calculate the maximum water capacity available (AWC) by subtracting the humidity in the PWP from the humidity value in the FC [57].

#### 4.4.3. Infiltration and Rate of Water Infiltration into the Soil

The accumulated water infiltration into the soil (AWI) was evaluated using the concentric ring infiltrometer method [58]. Readings were performed until constant infiltration values were obtained (five similar values). Readings were taken at the following time intervals: five repetitions of a minute; five repetitions of two minutes; five repetitions of five minutes; five repetitions of ten minutes; five repetitions of fifteen minutes; five repetitions of twenty minutes; and finally, intervals of thirty minutes, until the infiltration rate stabilized. The infiltration equations adjusted experimental data according to the mathematical models proposed by Kostiakov-Lewis. To obtain the time of basic infiltration rate (BIR), Equation (5) was used:

BIR = {[−0.001/[C × n × (n − 1)]}^1/(n−2)^ (5)

where BIR is the basic infiltration rate; n is the line slope, determined on the spot for each type of soil; and C is the constant showing the infiltrated blade in the first minute, in cm.

### 4.5. Data Analysis

For data analysis, the four field repetitions were used, and the variables were divided into five groups. The Yield components group was composed of plant height, the height of first pod insertion, number of pods per plant and thousand grains weight. The Physiological group was composed of the variables index SPAD, LAI, RWC, and Ψw. The group Root development was composed of the variables root area, average root diameter, root length density, and root dry matter. The group Soil physics was composed of the variables PR, TP, MP, mp, and Sd. The group Soil water by AWC, AWI, BIR, and SW in one, three, five, eight, and fifteen days after the rain.

In each variable group, it was applied to the principal component analysis (PCA) [59] through the nonlinear iterative partial least squares algorithm (NIPALS).

For each set of variables, the smallest possible number of components was sought that explained at least 70% of the total variability.

The five groups of variables scores were compared with the yield scores and plotted on scatter plots, considering the values of each replication.

From the scores of the principal components selected from each group of variables, the association with each component on the yield of soybean plants was assessed using Pearson’s linear correlation coefficient (*p* < 0.05).

## 5. Conclusions

The productive soybean performance is associated with yield components, soil physical-hydric properties, as well as physiological traits and plant root development.

The species in crop rotation in no-tillage system after 17 years of management interfere with soil physical and water characteristics, affecting physiological response and soybean yield. Sunn Hemp crop rotation in the spring season offers greater water stability to crops and provides better soybean yield in succession.

The joint assessment of variables that affect soybean yield provides more consistent data since these factors are generally correlated. The isolated evaluation of the variables can be insufficient to detect the limiting factors of the productivity, mainly when comparing different cover crops.

Our study proves that changes in the root growth environment promoted by soil management systems, especially by cover crops, are determinants of soybean yield. However, our results are from a single year of evaluation, and the degree of interference of these variables on soybean yield may vary between seasons, thus, more detailed studies, with results from year-to-year evaluations, are still necessary.

In addition to this factor, future research that better addresses the architecture and continuity of pores provided by crop rotation, and evaluations of gas exchange, fluorescence, and activities of stress enzymes in soybean plants may contribute to a better understanding of soil–plant relationships in long-term no-tillage.

## Figures and Tables

**Table 1:** Percentage explanation of the first major component of the variables yield components, physiological traits, root development, soil physics, and soil water.

Group of Variables	Explanation Percentage (%)
Yield components	71.62
Physiological traits	75.17
Root development	99.99
Soil physics	98.80
Soil water	97.70

**Table 2:** Chemical and granulometric analysis of soil managed in long-term no-tillage, at depth 0.00–0.20 m, with crop rotation with millet, sunn hemp, and occasional chiseling.

Spring Management	pH	P	OM	H + Al	Ca	Mg	K	Al	Sand	Silt	Clay
	CaCl_2_	mg dm^−3^		mmol_c_ dm^−3^					g kg^−1^		
Millet	4.6	47.5	27.1	54.8	7.4	2.3	5.5	8.8	110	245	645
Sunn hemp	5.0	43.0	30.9	46.3	8.2	3.3	4.9	4.0			
Chiseling	5.1	42.3	26.5	41.3	8.1	3.3	6.7	3.0			

pH: active acidity; P: exchangeable phosphorus; OM: organic matter; H + Al: potential acidity; Ca: exchangeable calcium; Mg: exchangeable magnesium; K: exchangeable potassium; Al: exchangeable aluminum; CaCl_2_: 0.01 M calcium chloride solution; mg dm^−3^: milligram per cubic decimeter; mmol_c_ dm^−3^: millimol charge per cubic decimeter; g kg^−1^: gram per kilogram.

**Table 3:** Crop rotation has been used since 1997, highlighting management and species cultivated in each agricultural year’s fall–winter (April to July), spring (September to November), and summer (December to March) seasons.

Year	Fall-Winter *	Spring	Summer
From 2003 to 2018	Triticale	Sunn Hemp	Soybean
		Millet	
		Fallow/chiseling **	

* Triticale was cultivated from 2003 to 2017 and maize was grown only in 2017. ** Soil chiseling was performed in 2003, 2009, 2013, and 2016. In treatments with chiseling, the soil remained fallow in the spring, that is, without cover crops, but with fall–winter and summer crops.

## Data Availability

Not applicable.

## References

[1] Palm C., Blanco-Canqui H., DeClerck F., Gatere L., Grace P. (2014). Conservation agriculture and ecosystem services: An overview. Agric. Ecosyst. Environ..

[2] Blanco-Canqui H., Ruis S.J. (2018). No-tillage and soil physical environment. Geoderma.

[3] Deuschle D., Minella J.P.G., Hörbe T.D.A.N., Londero A.L., Schneider F.J.A. (2019). Erosion and hydrological response in no-tillage subjected to crop rotation intensification in southern Brazil. Geoderma.

[4] Ferreira C.J.B., Tormena C.A., Severiano E.D.C., Zotarelli L., Betioli Júnior E. (2021). Soil compaction influences soil physical quality and soybean yield under long-term no-tillage. Arch. Agron. Soil Sci..

[5] Zhang Z., Peng X. (2021). Bio-tillage: A new perspective for sustainable agriculture. Soil Tillage Res..

[6] Elkins C.B. (1985). Plant roots as tillage tools. J. Terramechanics.

[7] Chen G., Weil R.R. (2010). Penetration of cover crop roots through compacted soils. Plant Soil.

[8] Calonego J.C., Raphael J.P.A., Rigon J.P.G., de Oliveira Neto L., Rosolem C.A. (2017). Soil compaction management and soybean yields with cover crops under no-till and occasional chiseling. Eur. J. Agron..

[9] Cresswell H., Kirkegaard J. (1995). Subsoil amelioration by plant-roots—The process and the evidence. Soil Res..

[10] Yunusa I.A.M., Newton P.J. (2003). Plants for amelioration of subsoil constraints and hydrological control: The primer-plant concept. Plant Soil.

[11] Kautz T., Lüsebrink M., Pätzold S., Vetterlein D., Pude R., Athmann M., Küpper P.M., Perkons U., Köpke U. (2014). Contribution of anecic earthworms to biopore formation during cultivation of perennial ley crops. Pedobiologia.

[12] Han E., Kautz T., Perkons U., Lüsebrink M., Pude R., Köpke U. (2015). Quantification of soil biopore density after perennial fodder cropping. Plant Soil.

[13] Naveed M., Moldrup P., Schaap M.G., Tuller M., Kulkarni R., Vogel H.-J., Wollesen de Jonge L. (2016). Prediction of biopore- and matrix-dominated flow from X-ray CT-derived macropore network characteristics. Hydrol. Earth Syst. Sci..

[14] Galdos M.V., Pires L.F., Cooper H.V., Calonego J.C., Rosolem C.A., Mooney S.J. (2019). Assessing the long-term effects of zero-tillage on the macroporosity of Brazilian soils using X-ray Computed Tomography. Geoderma.

[15] Zhang D.-W., Xiao Z.-J., Zeng B.-P., Li K., Tang Y.-L. (2019). Insect Behavior and Physiological Adaptation Mechanisms Under Starvation Stress. Front. Physiol..

[16] Pfeifer J., Kirchgessner N., Walter A. (2014). Artificial pores attract barley roots and can reduce artifacts of pot experiments. J. Plant Nutr. Soil Sci..

[17] Colombi T., Braun S., Keller T., Walter A. (2017). Artificial macropores attract crop roots and enhance plant productivity on compacted soils. Sci. Total Environ..

[18] Inagaki T.M., de Moraes Sá J.C., Tormena C.A., Dranski A., Muchalak A., Briedis C., de Oliveira Ferreira A., Giarola N.F.B., da Silva Á.P. (2021). Mechanical and biological chiseling impacts on soil organic C stocks, root growth, and crop yield in a long-term no-till system. Soil Tillage Res..

[19] Guedes Filho O., da Silva A.P., Giarola N.F.B., Tormena C.A. (2013). Structural properties of the soil seedbed submitted to mechanical and biological chiseling under no-tillage. Geoderma.

[20] Nunes M.R., Denardin J.E., Pauletto E.A., Faganello A., Pinto L.F.S. (2015). Effect of soil chiseling on soil structure and root growth for a clayey soil under no-tillage. Geoderma.

[21] de Moraes M.T., Debiasi H., Carlesso R., Franchini J.C., da Silva V.R. (2014). Critical limits of soil penetration resistance in a rhodic Eutrudox. Rev. Bras. Ciência Do Solo.

[22] Blanco-Canqui H., Wortmann C.S. (2020). Does occasional tillage undo the ecosystem services gained with no-till? A review. Soil Tillage Res..

[23] Abrahão G.M., Costa M.H. (2018). Evolution of rain and photoperiod limitations on the soybean growing season in Brazil: The rise (and possible fall) of double-cropping systems. Agric. For. Meteorol..

[24] da Silva G.F., Calonego J.C., Luperini B.C.O., Chamma L., Alves E.R., Rodrigues S.A., Putti F.F., da Silva V.M., de Almeida Silva M. (2022). Soil—Plant Relationships in Soybean Cultivated under Conventional Tillage and Long-Term No-Tillage. Agronomy.

[25] Hubert F., Hallaire V., Sardini P., Caner L., Heddadj D. (2007). Pore morphology changes under tillage and no-tillage practices. Geoderma.

[26] Osunbitan J.A., Oyedele D.J., Adekalu K.O. (2005). Tillage effects on bulk density, hydraulic conductivity and strength of a loamy sand soil in southwestern Nigeria. Soil Tillage Res..

[27] Deiss L., Kleina G.B., Moraes A., Franzluebbers A.J., Motta A.C.V., Dieckow J., Sandini I.E., Anghinoni I., Carvalho P.C.F. (2019). Soil chemical properties under no-tillage as affected by agricultural trophic complexity. Eur. J. Soil Sci..

[28] Sokolowski A.C., Prack McCormick B., De Grazia J., Wolski J.E., Rodríguez H.A., Rodríguez-Frers E.P., Gagey M.C., Debelis S.P., Paladino I.R., Barrios M.B. (2020). Tillage and no-tillage effects on physical and chemical properties of an Argiaquoll soil under long-term crop rotation in Buenos Aires, Argentina. Int. Soil Water Conserv. Res..

[29] Sun Y., Zhang N., Yan J., Zhang S. (2020). Effects of Soft Rock and Biochar Applications on Millet (*Setaria italica* L.) Crop Performance in Sandy Soil. Agronomy.

[30] Smith M.R., Reis Hodecker B.E., Fuentes D., Merchant A. (2022). Investigating Nutrient Supply Effects on Plant Growth and Seed Nutrient Content in Common Bean. Plants.

[31] Raven J.A., Smith F.A. (1976). Nitrogen assimilation and transport in vascular land plants in relation to intracellular ph regulation. New Phytol..

[32] Xiao X., Fan M., Wang E., Chen W., Wei G. (2017). Interactions of plant growth-promoting rhizobacteria and soil factors in two leguminous plants. Appl. Microbiol. Biotechnol..

[33] Daigh A.L.M., Dick W.A., Helmers M.J., Lal R., Lauer J.G., Nafziger E., Pederson C.H., Strock J., Villamil M., Mukherjee A. (2018). Yields and yield stability of no-till and chisel-plow fields in the Midwestern US Corn Belt. Field Crops Res..

[34] Zhao C., Gao J., Huang Y., Wang G., Zhang M. (2016). Effects of Vegetation Stems on Hydraulics of Overland Flow Under Varying Water Discharges. Land Degrad. Dev..

[35] Steponavičienė V., Bogužas V., Sinkevičienė A., Skinulienė L., Vaisvalavičius R., Sinkevičius A. (2022). Soil Water Capacity, Pore Size Distribution, and CO_2_ Emission in Different Soil Tillage Systems and Straw Retention. Plants.

[36] Pucciariello C., Perata P. (2021). The Oxidative Paradox in Low Oxygen Stress in Plants. Antioxidants.

[37] Siczek A., Lipiec J. (2011). Soybean nodulation and nitrogen fixation in response to soil compaction and surface straw mulching. Soil Tillage Res..

[38] Marcelo A.V., Corá J.E., Fernandes C., Martins M.D.R., Jorge R.F. (2009). Crop sequences in no-tillage system: Effects on soil fertility and soybean, maize and rice yield. Rev. Bras. Ciência Do Solo.

[39] Calonego J.C., Rosolem C.A. (2010). Soybean root growth and yield in rotation with cover crops under chiseling and no-till. Eur. J. Agron..

[40] Hama J.R., Kolpin D.W., LeFevre G.H., Hubbard L.E., Powers M.M., Strobel B.W. (2021). Exposure and transport of alkaloids and phytoestrogens from soybeans to agricultural soils and streams in the midwestern United States. Environ. Sci. Technol..

[41] Henry C., John G.P., Pan R., Bartlett M.K., Fletcher L.R., Scoffoni C., Sack L. (2019). A stomatal safety-efficiency trade-off constrains responses to leaf dehydration. Nat. Commun..

[42] Takahashi S., Badger M.R. (2011). Photoprotection in plants: A new light on photosystem II damage. Trends Plant Sci..

[43] do Rosário Rosa V., dos Santos A.L.F., da Silva A.A., Sab M.P.V., Germino G.H., Cardoso F.B., de Almeida Silva M. (2021). Increased soybean tolerance to water deficiency through biostimulant based on fulvic acids and *Ascophyllum nodosum* (L.) seaweed extract. Plant Physiol. Biochem..

[44] Abdallah N.A., Moses V., Prakash C. (2014). The impact of possible climate changes on developing countries: The needs for plants tolerant to abiotic stresses. GM Crops Food.

[45] Basche A.D., Delonge M.S. (2019). Comparing infiltration rates in soils managed with conventional and alternative farming methods: A meta-analysis. PLoS One.

[46] de Andrade Bonetti J., Anghinoni I., de Moraes M.T., Fink J.R. (2017). Resilience of soils with different texture, mineralogy and organic matter under long-term conservation systems. Soil Tillage Res..

[47] Soil Survey Staff (2014). Keys to Soil Taxonomy.

[48] Raij B.V., Andrade J.C., Cantarella H., Quaggio J.A. (2001). Análise Química Para Avaliação Da Fertilidade De Solos Tropicais.

[49] Teixeira P.C., Donagemma G.K., Fontana A., Teixeira W.G.T., EMBRAPA (2017). Manual de Métodos de Análise de Solo.

[50] Escobedo J.F., Gomes E.N., Oliveira A.P., Soares J. (2009). Modeling hourly and daily fractions of UV, PAR and NIR to global solar radiation under various sky conditions at Botucatu, Brazil. Appl. Energy.

[51] Watson D.J. (1947). Comparative Physiological Studies on the Growth of Field Crops: I. Variation in Net Assimilation Rate and Leaf Area between Species and Varieties, and within and between Years. Ann. Bot..

[52] Watson D.J. (1947). Comparative Physiological Studies on the Growth of Field Crops: II. The Effect of Varying Nutrient Supply on Net Assimilation Rate and Leaf Area. Ann. Bot..

[53] Jamaux I., Steinmetz A., Belhassen E. (1997). Looking for molecular and physiological markers of osmotic adjustment in sunflower. New Phytol..

[54] Ministério da Agricultura, Pecuária e Abastecimento (2009). Regras Para Análise de Sementes.

[55] Blake G.R., Hartge K.H., Klute A. (1986). Bulk Density. Methods of Soil Analysis: Physical and Mineralogical Methods.

[56] Smith K.A., Mullins C.E. (1991). Soil Analysis: Physical Methods.

[57] Reeve M.J., Carter A.D., Smith K.A., Mullins C.E. (1991). Water release characteristic. Soil Analysis: Physical Methods.

[58] Bernardo S., Mantovani E.C., Da Silva D.D., Soares A.A. (2019). Manual de Irrigação.

[59] Hair J.F., Black W.C., Babin B.J., Anderson R.E., Tatham R.L. (2009). Análise Multivariada de Dados.

